# Determination and Comparison of Short-Chain Fatty Acids in Serum and Colon Content Samples: Alzheimer’s Disease Rat as a Case Study

**DOI:** 10.3390/molecules25235739

**Published:** 2020-12-04

**Authors:** Lin-Xiu Guo, Yue Tong, Jue Wang, Guo Yin, Hou-Shuang Huang, Long Zeng, Ping Wang, Jun-Peng Li, Kai-Shun Bi, Tie-Jie Wang

**Affiliations:** 1 Shenzhen Key Laboratory of Drug Quality Standard Research, Shenzhen Institute for Drug Control, Shenzhen 518057, China; guolx1104@163.com (L.-X.G.); tongy26@163.com (Y.T.); wangjuepha@126.com (J.W.); ayinguoa@126.com (G.Y.); huanghoushuang21@163.com (H.-S.H.); kim19880220@163.com (L.Z.); wangping662@sina.com (P.W.); lali999@21cn.com (J.-P.L.); 2 School of Pharmacy, Shenyang Pharmaceutical University, Shenyang 110016, China

**Keywords:** short-chain fatty acids, determination, Alzheimer’s disease, LC-MS/MS, serum, colon content

## Abstract

Short-chain fatty acids (SCFAs) are the main microbial fermentation products from dietary fibers in the colon, and it has been speculated that they play a key role in keeping healthy in the whole-body. However, differences in SCFAs concentration in the serum and colon samples had attracted little attention. In this study, we have optimized the extract and analysis methods for the determination of ten SCFAs in both serum and colon content samples. Methanol and acetonitrile were chosen for extraction of SCFAs from serum and colon content samples, respectively. Biological samples were collected from Alzheimer’s disease rats treated by extract of *Schisandra chinensis* (Turcz.) Baill (SC-extract) were taken as research objects. The results showed that, the relative peak intensities of SCFAs in the colon content from all groups were quite similar, and the trend was identical in the serum samples. Compared with the values in humans, the ratio of ten SCFAs in rat’s colon was similar, while the percent of acetate in rat’s serum was significantly higher. For therapy of Alzheimer’s disease (AD), SC-extract decreased the concentration of butyrate, 3-Methyvalerate, and caproate in the serum samples towards the trend of normal rats. This study may help our understanding of how SCFAs are transported across colonic epithelium in healthy and diseased organisms.

## 1. Introduction

Alzheimer’s disease (AD) is a neurodegenerative disease that is characterized by progressive cognitive impairment. As a key factor in the process of learning and memory, cholinergic neurotransmission acetylcholine is correlated strongly with cognitive impairment in the disease [1]. Other demonstrated pathophysiological aspects of AD include amyloid beta (Aβ) peptide accumulates and the evoked pathological changes (abnormally phosphorylated tau protein and inflammatory affection) [2]. But the complex pathogenesis of AD remains unclear [3]. Recently, it has been hypothesized that AD may be associated with the dysbiosis of gut microbiota and their metabolite [4]. As the most important metabolism of gut microbiota, short-chain fatty acids (SCFAs) had also been demonstrated to inhibit Aβ aggregations [5]. As examples, valeric acid, and butyric acid were observed to potently interfere with initial Aβ_1–40_ protein–protein interactions at an SCFA: Aβ molar ratio ranging from 1:1 to 4:1 [5].

SCFAs, which are the main microbial fermentation products from dietary fiber, are speculated to play a key role in energy homeostasis [6], confer anti-inflammatory [7], immunomodulatory systemic effect [8], microbiota–gut–brain crosstalk [9], etc. For humans, SCFAs are produced in the gut from bacterial degradation of carbohydrates and proteins at a total concentration of 50 mM–200 mM [10,11,12]. Acetate (C2), propionate (C3), and butyrate (C4) are the major anions that formed at a relatively constant ratio of about 60:25:15, respectively [11,13,14].

All the SCFAs produced in the colon are employed through the two following pathways: (1) 95% of total SCFAs are absorbed by colonic cells to provide energy in the form of ATP or to travel via the basolateral membrane into systemic circulation; (2) ±5% of total SCFAs are lost in fecal excretion [9]. The complex routes of SCFAs lead to a biological gradient across the various down-stream tissues and differences of detected concentrations in different sources of biological samples [12]. For example, a previously published study [15] identified that acetic acid, propionic acid, and butyric acid were the main detected SCFAs in mice fecal samples, and isobutyric acid was also detected in the serum samples in addition to the ones in feces. This data indicated that the concentrations of luminal SCFAs do not always reflect the concentrations of absorbed SCFAs. Hence, paying attention to the differences and relationships between the serum SCFAs concentration and colon SCFAs concentration may explain the pivotal role of SCFAs in physical health.

Another situation worth watching is that, much attention has been paid to colonic or fecal SCFAs in the previous studies, but much less attention to the relationship between colonic SCFAs concentrations and levels in the systemic circulation (such as serum) [6]. Two criteria searches were performed in the Pubmed database by utilizing the following key terms: (1) “Serum” or “plasma” in the title/abstract coupled with “SCFA” in the title/abstract; (2) “colon” or “colonic” or “fecal” or “feces” in the title/abstract coupled with “SCFA” in the title/abstract. As a result, search 1 yielded a list with 317 studies, and search 2 yielded 811 studies. The utility of SCFAs quantitation method used for the colon content may be limited because of the high protein, but low concentrations of SCFAs in the serum samples. Up to now, no method has been reported for the comparison of SCFAs in the colon content samples and serum samples.

To compare the colon and serum levels of SCFAs, AD animal models were established through a direct infusion of oligomeric Aβ (1–42) into the CA1 subregion of the rat hippocampus. Meanwhile, the various extracts from traditional Chinese medicine provided valuable materials in the preventive or treatment of several human diseases; among that, extract of *Schisandra chinensis* (Turcz.) Baill (SC-extract) was a noticeable pharmaceutical ingredient of AD for cognitive improvement of sesquiterpene compound (α-isocubebenol) [16], lignan compound (total [17], or single lignan [18]), and polysaccharide compound [19]. Thus, this study measured and compared the SCFAs concentration in the colon content and serum basing on the samples collected from the Aβ_1–42_ peptide-induced AD model rats, which were treated with or without SC-extract.

## 2. Results and Discussion

### 2.1. SC-Extract Regulated the Learning and Memory Impairment Induced by Aβ_1–42_ in Rats

AD is a neurodegenerative disorder characterized by memory loss. Behavioral experiment is key for examining learning and memory ability in animal experiments, among which the Morris water maze (MWM) test is the most classical method. According to the previous reports, the water maze experiment includes two phases: (1) Response acquisition and space exploration experiment; (2) probe and extinction trials. In the first phase, the hidden platform and the space indicator are provided to examine the time required for the animal to find the escape platform within a certain range (the diameter of the pool), and then evaluate its space cognitive ability by using hidden platform task. After the positioning and navigation experiment continued for a certain period, the space exploration experiment was used to investigate the learning and memory abilities of the animals, by retaining the marks, but not providing an escape platform.

**Figure 1:**
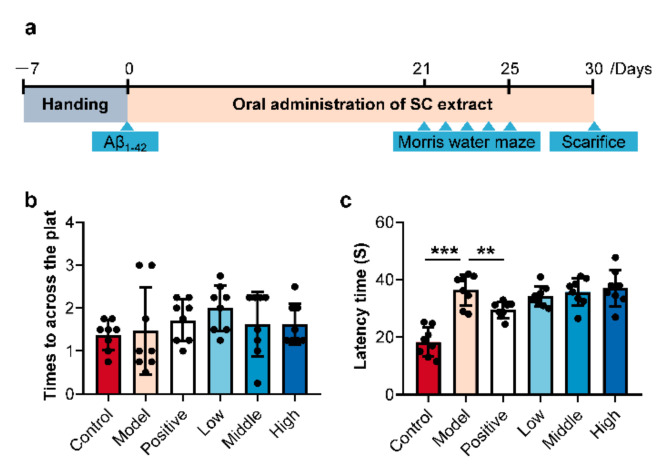
The schedule of animal experiment in this research (**a**) and the results of behavioral testing included times to across the platform (**b**) and escape latencies (**c**) in probe and extinction trials (phase 2) of Morris water maze test (*n* = 8–11 per group. * *p* < 0.05; ** *p* < 0.01; *** *p* < 0.001).

In the present study, the AD model was obtained by injecting Aβ_1–42_ peptides into the brain via intrahippocampal injections. Donepezil, which is a well-absorbed acetylcholinesterase inhibitor (AChEI) with a longer half-life and minimum interaction with other drugs [20], was chosen as the positive drug for its efficacy on cognitive function across the mild, moderate, and severe stages of AD [21,22]. MWM test was used to examine the spatial cognitive performance of animals. On the first day of phase 1(day 21), a high dose of SC-extract significantly attenuated the effects of Aβ_1–42_ on escape latencies and swimming distances (escape latency: *p* = 0.0248; swimming distance: *p* = 0.0068; Figure S1a,e. In the meantime, the low (100 mg/kg) and middle dose (200 mg/kg) of SC-extract decreased the average distances to the platform (low: *p* = 0.0205; middle: *p* = 0.0159; Figure S1i). However, on the second and third days of phase 1 (day 22 and 23), all drug-treated groups did not show a significant effect (Figure S1b,c,f,g,j,k). On the last day of phase 1 (day 24), positive drug (0.45 mg/kg donepezil), low and middle dose of SC-extract significantly attenuated the swimming distances of the Aβ_1–42_-treated rats (Figure S1h). In phase 2, positive drug reduced the higher escape latency of model rats (control vs. model: *p* = 0.000; model vs. positive: *p* = 0.004; Figure 1b,c). Taken together, these various observations suggested that SC-extract improved the rats’ cognitive ability to find the hidden platform in the initiation of behavioral test, while did not stably promote the memory ability of Aβ_1–42_-treated rats.

### 2.2. SC-Extract Regulated the Inflammation and Pathological Indexes of AD Rats

Reduction of acetylcholinesterase (Ach) release and generation of amyloid plaques and tau phosphorylation in the brain are key events in the pathogenesis of AD [23,24]. It once appeared as described above, a systemic inflammatory response will be triggered, along with compromising brain functions of learning and memory. Furthermore, comparing to amyloid-negative patients, amyloid-positive patients showed an upregulation trend of several classical pro-inflammatory markers (including TNFα, IL-1β, and IL-6) and a reduced blood level of the anti-inflammatory cytokines (such as IL-10) [25]. In contrast, downregulation of IL-10 was considered as a risk factor for AD in an earlier conclusion, while IL-10 levels were increased in the AD brains, paradoxically [26].

**Figure 2:**
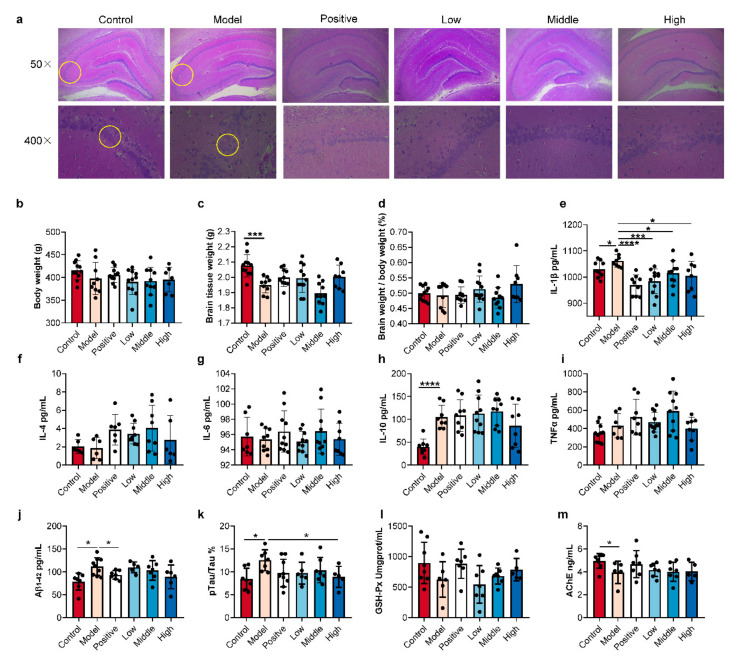
The extract of *Schisandra chinensis* (Turcz.) Baill (SC-extract) ameliorated the Aβ-induced memory impairment (**a**), brain tissue weight (**b**–**d**), inflammatory cytokines (**e**–**i**), and Alzheimer’s disease (AD)-associated indexes (**j**–**m**). Data are presented as means ± SD (*n* = 8–11 per group and the solid circles mean samples. Kruskal-Wallis’ test, * *p* < 0.05, ** *p* < 0.01, *** *p* < 0.005, **** *p* < 0.001).

To assess the regulation of SC-extract on AD rats, we evaluated histological analysis, brain weight determination, anti-inflammatory assay, and AD biomarkers (Figure 2a–m). Brain perfusion and HE staining were performed as previously [18] (Figure 2a), and the number of surviving neurons was dramatically lower in the granule cell layer of the CA region of the AD model group than in the control group. While in the SC-extract treated rats, especially the low dose group, the surviving neurons were much more. For the brain weights, significant loss was observed in the model rats comparing with the healthy control ones (Figure 2c). Then, IL-1β levels in model rats were much higher than in control rats, while the SC-extract and positive drug significantly reduced the indexes (Figure 2e). Meanwhile, IL-10 levels (which were significantly higher in model rats than in control rats) had not been regulated by SC-extract oral administration (Figure 2h). However, no significant alterations in body weight, ratio of brain weight in body weight, or level of inflammation indexes, including IL-4, IL-6, and TNF-α, were found in the comparisons between the model and the other groups (Figure 2b,d,f,g,i). The last focused indexes were the levels of Aβ_1–42_, pTau/Tau, AchE, and GSH-px in brain tissue. As a result, significant increases in Aβ_1–42_ level and ratio of pTau/Tau were observed in AD compared with control rats, accompanied by a decrease in AchE level. A positive drug and high dose of SC-extract regulated the Aβ_1–42_ level and pTau/Tau ratio, respectively (Figure 2j,k,m).

### 2.3. Optimization of Extraction and Derivatization Method for SCFAs Determination

Mounting evidence suggests that AD is linked to a disorder of gut microbiota, which may promote chronic inflammation and anabolic resistance [27]. As the key metabolite, SCFAs play a key role as molecules at the interface between the activity of gut microorganisms and host metabolism. The sample sources for SCFAs included fecal [28], colon content [29], serum [30], bronchoalveolar lavage fluid [31], and others [32]. Among all, the colon content and serum, as the SCFAs main produced location and the key pathway of transport, are very important for the analysis of SCFAs. Different kinds of samples required different extraction process, thus optimization of SCFAs extraction was firstly performed (Table S1).

To optimize the extraction method of SCFAs, serum samples were processed according to the above sample processing methods, and the response values of SCFAs in QC samples were detected using the chromatography and mass spectrometry conditions in the materials and methods. The results showed that all SCFAs except acetic acid in the serum were shown at a low level of mass spectrometry responses (≤10^5^). So, we consider increasing the relative volume of serum extraction in the derivatization system and the test solution. Firstly, 40 μL, 60 μL, and 100 μL serum QC samples were, respectively, added into a two-fold volume of methanol for extraction and made the final volume of 1 mL after derivatization. As a result, the ratio of 60 μL: 120 μL (serum samples: methanol) was chosen because of its effective mass spectrometry response and loss of impurity. After a vortex with methanol for 10 min, 100 μL of the supernatant was collected as a derivatized sample. Then, 100 μL of derivatization reagent (see Materials and methods) was added to establish the chemical derivatization reaction system. The system was incubated at 40 °C for 30 min and made up to 1 mL with methanol, and the test solution was collected from the supernatant after centrifugation at 15,493× *g* for 10 min.

Several pre-processing methods have been used for the extraction of SCFAs in the serum samples, including 2-nitrophenylhydrazine direct derivatization followed by diethyl ether extraction and HPLC-UV analysis, which need strict quantitative process and result in poor peak separation [33], hollow fiber supported liquid membrane extraction followed by acidified and GC-MS analysis with only 6 SCFAs determined [34], and anhydrous ether extraction followed by N, O-bis(trimethyl-silyl)-trifluoroacetamide derivatization and GC/MS analysis which also need dehydration to progress by sodium sulfate [15]. However, insufficient quantification of trace SCFAs and/or strict quantitative processes limit the applicability of these methods. Thus, in the present study, protein precipitation methods with characteristics of less time-consuming and simple operation were used for the pretreatment of serum sample.

For colon content, the appropriate ratio of freeze-dried samples (weight) and 95% acetonitrile (volume) was optimized at 40 mg: 960 μL in our previous study. The mass spectrometry response of SCFAs obtained from colon content was about at a level of 10^6^ or 10^7^. Therefore, the derivatization reaction process of SCFAs in the colon content was as follow: 40 mg of freeze-dried samples and 960 μL of 95% acetonitrile were added into a tube vortexed for 10 min at 4 °C, 40 μL of the supernatant was collected after centrifugation at 15,493 g for 10 min. For derivatization, 40 μL of derivatization reagent was added into the 40 μL of supernatant, followed by incubating at 40 °C for 30 min. Then the solution system was made up to 1 mL with prechilled 95% acetonitrile. The supernatant after centrifugation at 15,493 g for 10 min was collected as the test solution.

And then, the calibration curves determined for all SCFAs in both serum and colon content samples showed that the coefficient of determination (R^2^) was greater than 0.996 (see Figures S5 and S6, and Table S2). Method validation including linearity range, repeatability, precision, recovery, derivatization reaction yield, and matrix effect according to published reports [31]. The CV values of precision obtained from repeatability, intra- and inter-day precision, and recovery were satisfactory (Table S3). These results suggest that optimized extraction, derivatization, and UPLC-TQ/MS method for SCFAs methods can be used for the determination and relative quantification of SCFAs in the colon content and serum sample without stable-isotope labeled standards.

### 2.4. Calculated and Compared the SCFAs Concentrate in Serum and Colon Content Samples

Recently, it has been hypothesized that a dysbiosis of microbes is closely associated with a high incidence of AD. As the most important metabolism of gut microbiota, SCFAs had also been demonstrated to inhibit Aβ aggregations [5]. SCFAs can directly activate G-coupled-receptors (in lumen), inhibit histone deacetylases (in lumen), serve as energy substrates (in the colonic epithelium), and provide an energy substrate for hepatocytes (through reaching systemic circulation) [35]. A previous study has demonstrated that a high dose of valeric acid, butyric acid, and propionic acid is capable of interfering with the aggregation of Aβ_1–40_ and/or Aβ_1–42_, and valeric acid could inhibit the conversion of monomeric Aβ_1–40_ and Aβ_1–42_ into Aβ fibrils [5].

**Figure 3:**
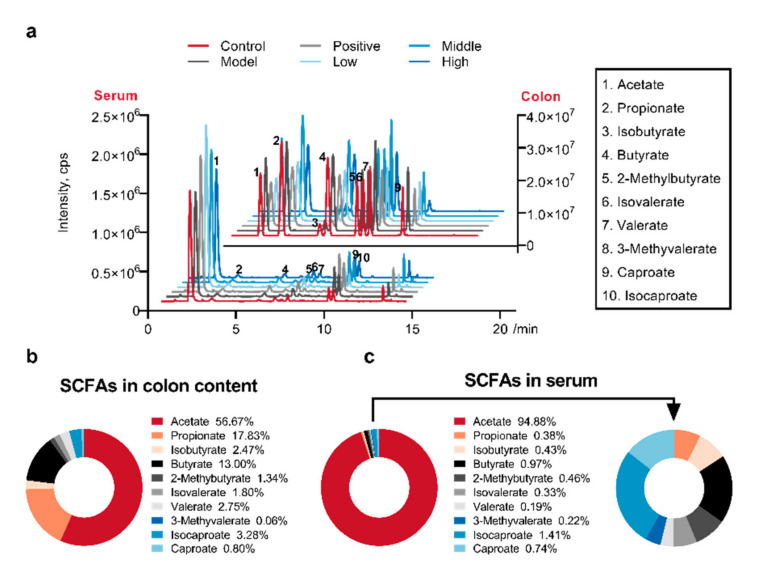
Diversity of short-chain fatty acids (SCFAs) integral distribution in the serum and colon content samples. (**a**) The average intensity of derivatived SCFAs were calculated for serum and colon content samples of different groups. (**b**,**c**) The average percent compositions of SCFAs in the colon (**b**) and serum (**c**) samples were calculated for all groups and shown in doughnut charts.

With the optimized methods, the concentration of SCFAs in the serum and colon content were determined. The relative peak intensities of SCFAs in the colon content from all groups were quite similar, and the trend was identical in the serum samples (Figure 3a). In the colon content (top right of the figure), most of the SCFAs in all groups showed high concentration levels except isobutyrate. While in the serum (bottom left of the figure), most of the SCFAs in all groups showed low concentration levels except acetate. This phenomenon indicated that a high dose of colonic SCFAs did not represent a high concentration of serous SCFAs. It was obvious that response levels of SCFAs in the serum (10^5^–10^6^) were much lower than that in the colon content (10^7^). Meanwhile, the treatment of AD and SC-extract exhibited much less obvious differences in the colon and serum SCFAs, respectively. This result was demonstrated by the average percent in the colon and serum samples of all groups (Figure 3b,c). According to the previous study, acetate, propionate, and butyrate are the three major species with a total concentration of around 100 mmol∙L^−1^, and the sum concentration of other SCFAs is about 10 mmol∙L^−1^ [12,36]. In our study, the sum concentrate ratio of three major species to the others is 87.5: 12.5 in the colonic lumen, which is similar in humans. Furthermore, the acetate, propionate, and butyrate were at a molar ratio of 57:22:21 in the human colonic lumen and 71:21:8 in portal blood in a previous study [24]. In this paper, the average value of this ratio is 65:20:15 in the colonic lumen, and 98.6:0.4:1 in blood, respectively. The ratio in the colon was similar in humans, while the percent of acetate in the serum was significantly higher.

**Figure 4:**
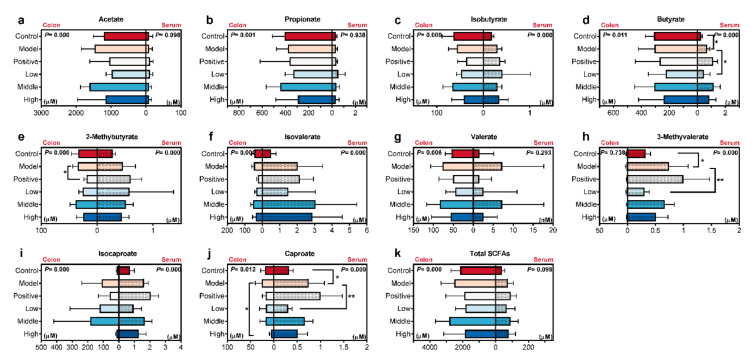
Concentrate on the ten SCFAs from the two kinds of samples in different groups (**a**, acetate; **b**, propionate; **c**, isobutyrate; **d**, butyrate; **e**, 2-methylbutyrate; **f**, isovalerate; **g**, valerate; **h**, 3-methyvalerate; **i**, isocaproate; **j**, caproate; **k**, total SCFAs). For every single plot, the left part showed the SCFAs concentrate in the colon content, and the right part showed the concentrate in the serum. Two red colors mean groups untreated with drugs, and the four blue colors mean groups treated with drugs (donepezil or SC-extract). Mean of variables in different groups were compared by Kruskal-Wallis’ test (*n* = 8–11 per group. * *p* < 0.05, ** *p* < 0.01, *** *p* < 0.005, **** *p* < 0.001).

Afterward, the concentrates of the ten SCFAs from the two kinds of samples in different groups were shown in Figure 4 and Table S4. For every determined SCFA, the normality of data was firstly assessed, and then the non-parametric ANOVA method (Kruskal-Wallis’ test) was chosen for comparison. On the left of every plot, the concentrate in the colon content from different groups was compared with each other. 2-methybutyrate in the positive group and caproate in the high SC-extract treated group revealed a significant decline compared with the AD model rats, respectively (Figure 4e,j). Different from the results in the colon content, the ANOVA analysis demonstrated that serum levels of butyrate, 3-Methyvalerate, and caproate were significantly elevated in AD model rats compared with ones in the control group (right of Figure 4d,h,j). Furthermore, these three SCFAs were significantly decreased toward the level of the control group by a low dose of SC-extract. However, no significant difference in other detected SCFAs was observed in the comparisons between the model and the other groups for the colon content and serum samples (Figure 4a–c,f,g,i), and the same for the total concentration (Figure 4k). Taken together, different trends for SCFAs levels were found in the colon content and serum. After treated with SC-extract in the dose of 100 mg/kg, the colonic barrier and SCFAs absorbability were regulated towards the trend of normal rats (control group).

The rate of SCFAs uptake across the cell membrane would increase with the lipid/water partition coefficient in conventional wisdom [37]. However, the recent analysis indicated the effect of transporting proteins (including SLC26A3, MCT1, and SLC16A1) [11] become increasingly obvious in the transport of SCFAs. The two most important reported SCFAs transporters families are monocarboxylate transporter (MCT) and solute carrier (SLC) family [38,39]. These transport systems operate depending on the transmembrane H^+^ gradient, Na^+^ gradient, and HCO^3−^ gradient [38]. Except for acetate, whose concentration in the serum may be influenced by various transformed sources in the whole body, other SCFAs were primarily generated by the bacteria reside in the colon and transported to the blood. So far, the influence of AD on the levels of SCFAs in the serum and colon content has not been clear. In the present study, six SCFAs (including acetate, propionate, isobutyrate, isovalerate, valerate, and isocaproate) showed similar levels in the colon content and serum samples from different groups, respectively. For the total concentrate of ten tested SCFAs, the same result was observed. These indicted that the homeostasis of these SCFAs was not influenced by the injection of Aβ_1–42_ peptide and oral administrator of SC-extract. Unlike our results, lower levels of acetate and propionate were determined in feces and hippocampus samples of Aβ_1–42_-induced mice in a previous study [40]. However, the different sample sources make it hard to compare and explain the differences in results.

**Figure 5:**
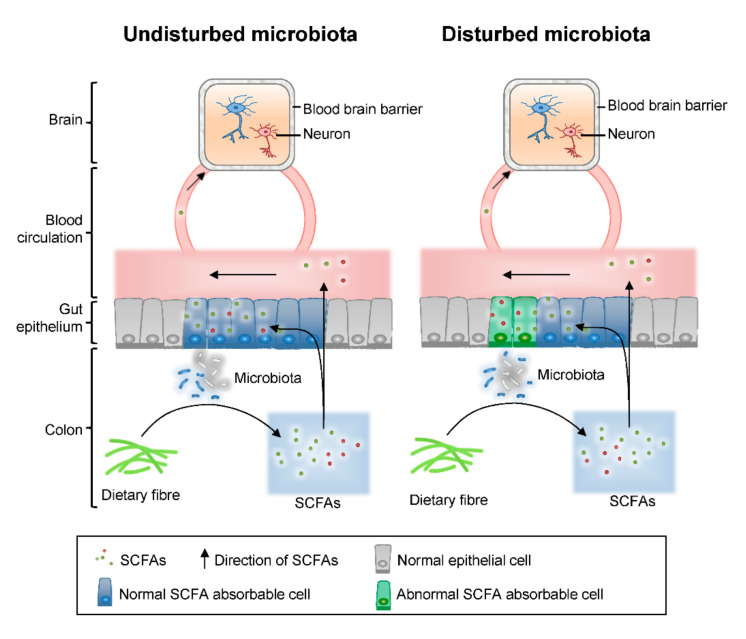
The pathological changes induced by Aβ_1–42_ injection may lead to the disturbance of the colonic barrier.

On the other hand, for butyrate, 3-methyvalerate, and caproate, more absorption was observed in model rats, even though the production of SCFAs in the colon lumen kept stably. Some evidence indicates that butyrate is capable of exerting its effects on glycolipid metabolism abnormalities and disease via the gut-brain axis [41]. However, there is no clear conclusion on the effect of SCFAs on brain learning and memory function. We speculate that the abnormal absorption in the present study may be associated with a disturbance of the colonic barrier and expression transporting proteins induced by Aβ_1–42_ injection (Figure 5). To reveal the detailed influence of AD on the SCFAs transportation, further analysis should be developed with feces and hippocampus samples for the determination and quantification, or intestinal barrier function and SCFAs transporter associated gene expression should be analyzed.

For SC-extract, the lowest drug dose offered the most marked pharmaceutical effect comparing with the middle and high dose groups. The possible reason was that the dissolution of bioactive compounds, such as lignans and polysaccharides, was inhibited in the higher dose of oral solution. On the previous study, polysaccharides from *Schisandra chinensis* fruits showed immunomodulatory activities by enhancing the secretion of IL-1β and TNF-α from RAW264.7 cells [42] and increasing the TNF-α levels in blood serum of tumor-bearing mice [43]. In our study, SC-extract increased the IL-1β levels in the serum of AD rats, while had no effect on the TNF-α levels. In addition, it is still a problem whether altered serous SCFAs levels can affect the AD-associated disorder by traversing the BBB (blood-brain-barrier), even though valeric, butyric, and propionic acid have been demonstrated to interfere with the formation of Aβ aggregates [5]. Meanwhile, the limited correlation between SCFAs concentrate (in the serum and colon content) and the above detected measured (inflammation and pathological) indexes may be indicted that SCFAs would interact very weakly with AD-associated pathological changes (Figure S2). Thus, future studies are worth pursuing to clarify the transportation process of SCFAs from colon lumen to systemic circulation, and its role in the associated mechanisms of SC-extract treating Alzheimer’s disease.

## 3. Materials and Methods

### 3.1. Animal Experimental Design

Male Wistar rats (150 ± 20 g) were obtained from the Guangdong Medical Laboratory Animal Center (Foshan, China). All animals were housed under clean grade conditions at the laboratory animal center of the Shenzhen Institute for Drug Control (Shenzhen, China). The environment was maintained at 44–65% humidity and a 12-h light/dark cycle at 22–26 °C. All animal experiment protocols were approved by the Animal Ethics Committee of Shenzhen Institute for Drug Control (21 March 2019). For this study, the rats were randomly assigned to additional groups as follows: A sham-operated control group treated with saline (control, *n* = 10); an Aβ_1–42_ peptide-treated group treated with saline (model, *n* = 9); an Aβ_1–42_ peptide-treated group treated with 0.45 mg/kg donepezil (positive, *n* = 10); three Aβ_1–42_ peptide-treated group treated, respectively with low (100 g/kg, *n* = 11), middle (250 g/kg, *n* = 10) and high (500 g/kg, *n* = 8) dose of SC-extract. Saline, donepezil, and SC-extract were taken via intragastric administration and continued for 30 days. The scheme of animal experiments was shown in Figure 1a.

To induced AD model, the oligomers Aβ_1–42_ peptides were prepared by dissolved amyloid β protein fragment 1–42 in sterile water at a concentration of 1 mM and incubated at 37 °C for four days [44]. Then, 30 rats were randomly allocated, and stereotaxically injected intra-hippocampally with pre-aggregated Aβ_1–42_ peptides after seven handing days (Figure 1a) [45]. The MWM test, which included the acquisition phase (4 days) and probe trial (1 days), was used as described to assess the spatial reference learning and memory of all rats [46,47]. The parameters were set as follows: Diameter of the circular pool, 120 cm; the temperature of the water, 25 ± 2 °C; diameter of the colorless platform, 14 cm. In the acquisition phase, all animals were trained four trials per day the and 60 s were given to find the escape platform. In the probe trial, the platform was taken out, and the time spent in the target quadrant was recorded to assess spatial memory.

At the end of the experiment, the right-brain tissue was obtained for histopathological examination by staining with hematoxylin and eosin (H&E). Left-brain tissue was collected to measure the levels of Aβ_1–42_, p-Tau, Tau, AChE, and GSH-Px. Serum samples were collected via coagulation and centrifugation to measure inflammatory cytokines (IL-1β, IL-10, IL-6, IL-4, and TNFα) and SCFA analysis. After ligating both ends of the colon and freezing the whole tissue, colon content of all groups were collected following by splitting the colon wall. The colon content samples were freeze-dried and stored at −80 °C for SCFA analysis.

### 3.2. Histopathologic Analysis and ELISA Assay

Brains from different groups were perfused for histopathological examination by staining with hematoxylin and eosin (H&E) as described previously [48]. At the end of the animal experiment, the rats were anesthetized with chloral hydrate (i.p.). The right brain was removed quickly and immersed in 4% paraformaldehyde for two weeks, and then cut to neighboring serial sections in the thickness of 10-μm and stained on Symphony Staining System (Roche, AZ, USA). Histological specimens were examined in light microscopy (Lecia, Wetzlar, Germany).

Aβ_1–42_, p-Tau, Tau, AChE, and GSH-Px in brain homogenates were determined using commercially available ELISA kits (Covance, Princeton, NJ, USA) according to the manufacturer’s instructions. Inflammatory cytokines (IL-1β, IL-10, IL-6, IL-4, and TNFα) in the serum samples were determined using commercially available ELISA kits (Covance, Princeton, NJ, USA) according to the manufacturer’s instructions.

### 3.3. Chemicals and Reagents

SCFAs standards including acetate, propionate, isobutyrate, butyrate, 2-methylbutyrate, isovalerate, valerate, 3-methyvalerate, isocaproate, and caproate were purchased from Sigma-Aldrich (St. Louis, MO, USA). Derivatization reagents including 3-nitrophenylhydrazine hydrochloride (3-NPH), 1-ethyl (3-dimethyllaminopropyl) carbodiie hydrochlide (EDC·HCl), and pyridine were purchased from Sigma-Aldrich (St. Louis, MO, USA). HPLC grade acetonitrile (ACN) and methanol (MeOH) were obtained from Merck (Darmstadt, Germany). Formic acid (99%) was purchased from Sigma-Aldrich (St. Louis, MO, USA). Ultrapure water was obtained by filtration of distilled water using a Milli-Q system (Millipore, MA, USA).

*Schisandra chinensis* (Turcz.) Baill was purchased from Zhongshan Jianhe Chinese Medicine Pieces Co., Ltd. and identified by Dr. Shuhong Wang. Then, 1 kg of the medical powder (60 mesh) was extracted three times by heat-reflux with 10 L of 60% ethanol for 30 min each. The supernatant of the combined extract was concentrated to get SC-extract.

### 3.4. Extraction of SCFAs

For the colon content samples, SCFAs were extracted according to our previous experiment as follows: Approximately 40 mg of lyophilized colon contents were precisely weighed, and 960 μL of 95% acetonitrile were added, followed by vortex mixing for 10 min to extract the SCFAs. The suspensions were centrifuged for 10 min at 15,493× *g* and 4 °C.

For the serum samples, SCFAs were extracted by adding 200 mL methanol to 100 mL serum sample following by a vortex for 1 min. Then, the suspensions were centrifuged for 10 min at 2292× *g* and 4 °C.

### 3.5. Method of 3-NPH-Based Derivatization

SCFAs in the serum samples and colon contents were treated with a chemical derivatization method as previously described, with some modifications [49].

Two derivative reagents (2 mL) were prepared, respectively. Reagent 1: Constituted of 1 mL of 200 mmol/L 3-NPH, 200 L of 600 mmol/L EDC, and 800 L 7.5% pyridine and prepared in 30% acetonitrile. Reagent 2: Constituted of 1 mL of 100 mmol/L 3-NPH, 200 L of 300 mmol/L EDC, and 800 L 3.75% pyridine and prepared in 30% acetonitrile. The whole process was performed in an ice bath, and all reagents were fresh.

The SCFAs extraction was mixed with derivative reagent at the ratio of 1:1 (*v*/*v*; 200μL in total). Reagent 1 was used for the colon content samples, and reagent 2 was used for the serum samples. Afterward, the mixture was permitted to react in a dry, warm bath at 40 °C for 30 min. After the reaction, the mixture was cooled down at 4 °C and finally diluted to 1 mL with 30% cooled acetonitrile. Before injection, the mixed system was centrifuged at 20,627× *g* (4 °C) for 10 min, and the supernatant was used for LC-MS analysis.

### 3.6. Instrumentation

UPLC-MS/MS analysis was performed using ACQUITY UPLC H-Class system (Waters, Milford, MA, USA) and XEVO TQ-S mass spectrometer equipped with electrospray ionization (ESI) prob (Waters, Milford, MA, USA). Chromatographic separation was performed on a Waters Acquity BEH C18 column (2.1 × 50 mm, 1.7 m). The mobile phases were composed of 0.01% formic acid in water (*v*/*v*) (A) and 0.01% formic acid in acetonitrile (*v*/*v*) (B).The mobile phases were eluted at 0.30 mL/min with the gradient as follows: 0–3 min 15% B; 3–11 min 15–45% B; 11–12 min 55–99.9% B; 12–12.1 min 99.9–15% B; re-equilibrate at 15% B for 2 min. The column temperature was maintained at 40 °C, and the injection volume was 5 μL. The MS/MS detection was performed via electrospray ionization source in the negative mode (ESI^−^). The parameters of the mass spectrometer included a capillary voltage of 2.7 kV; a cone energy of 27 V; a desolvation temperature of 350 °C; desolvation gas of 600 L/Hr, and cone gas of 150 L/Hr. Multiple Reaction Monitor (MRM) conditions acquisition modes were optimized in MsaaLynx V4.1 software for the determination of derivatived SCFAs according to the published reports [49,50], and summarized in Table S5. All the peaks were integrated with a default peak-to-peak amplitude and one time of smooth in a window size of ±2. The peak detect baselines were set as follows: Join valleys if peaks resolved to 5.00% above baseline; reduce peak tailing until trailing edge is no more than 100.00% wider than the leading edge; raise baseline by no more than 0.00% of peak height. The peak separation was limited by the parameters as follows: Draw vertical if peaks resolved to 90% above baseline, detect shoulder peaks if the slope is less than 70.00% of maximum. The response threshold was above 3.00% for the relative area and 3000.00 for the absolute area. For each SCFA showed a single chromatographic peak in the profile, the MRM peaks were identified by their stable retention time.

### 3.7. Calibration Curve and Method Validation

Quality control (QC) samples from serum samples and colon contents were prepared from each biological sample to ensure broad metabolite coverage. Calibration standard solutions were prepared according to the peak areas of derivatived SCFAs in QC samples.

For the colon content samples, the QC samples were prepared by the mixed working solutions with 2-Methybutyrate, Valerate, and Caproate 3.9, 500, and 1000 nM, and other SCFAs 3.9, 1000, and 2000 nM as LQC (low-concentration quality control), MQC (medium-concentration quality control), and HQC (high-concentration quality control). For the serum samples, the QC samples were prepared by the mixed working solutions with Acetate 4.88, 78.125, and 625 nM, and other SCFAs 0.0049, 0.19, and 12.5 nM as LQC, MQC, and HQC.

Method validation including linearity range, repeatability, precision, recovery, derivatization reaction yield, and matrix effect according to published reports [31].

The matrix effect and the efficiency of the MRM method were shown with the chromatographic charts of a representative sample, control solvent, and blank solvent in Figure S3 (colon content sample) and Figure S4 (serum sample). The chromatographic peak area was integrated automatically. For the colon content sample, the potential interfering agents included the peaks at 1.80 min, 3.00 min, and 7.38 min. The first two peaks could not be integrated, and the area of peak at 7.38 min in control solvent was just 0.53% of this peak in the representative sample. For the serum sample, the only potential interfering agent was the peaks at 1.80 min in the channel of acetate, and the area was 4.7% of this peak in the representative sample. These data proved that the matrix effect was insignificant.

### 3.8. Data Analysis

The raw metabolomics data were deposited at the EMBL-EBI MetaboLights database [51] (https://www.ebi.ac.uk/metabolights/index) under projects MTBLS2146 (method validation) and MTBLS2088 (determination of SCFAs in the serum and colon content samples). Results of animal experiment and ELISA assays were presented as mean ± SD (calculated by Excel 11.0) and showed in bar plots with scatters (Figure 1b,c, and Figure 2b–m) by GraphPad Prism 8.0 (GraphPad Software, Inc., La Jolla, CA, USA). The average intensity of derivatived SCFAs in the serum and colon content samples from different groups were calculated in Excel. Afterward, the average values were imported into GraphPad Prism 8.0 to visualize in a multiple line chart (Figure 3a). The percent compositions of the ten detected SCFA in every sample were calculated firstly, and then the average value of percentages in all the measured samples was calculated according to the different sample types, as the data for drawing the donut plots (Figure 3b,c). For all statistically significant differences, ANOVA or Kruskal-Wallis tests were used for multiple comparisons, and student’s t-tests were used for two groups by version 24.0 (SPSS Inc., Chicago, IL, USA). Differences were noted as significant at *p* ≤ 0.05.

## 4. Conclusions

In this paper, we optimized the extraction process and determination method of SCFAs extracted from serum and colon content samples. The methods were then applied to determine the changed levels of ten SCFAs in samples collected from SC-extract treated AD rats. Our results indicated that regulation of colonic SCFAs levels may not induce a similar trend for serous SCFAs. In addition, SC-extract treatment decreased the significantly up-regulated butyrate, 3-Methyvalerate, and caproate in the serum sample of AD rats. The optimized method here can be extended for the determination and comparison of SCFAs levels of serum and colon samples derived from different patients or animal models, and would be helpful for understanding the complex interplay between SCFAs and host’s disease.

## Supplementary Materials

The following are available online, Figure S1. Effects of the extract of *Schisandra chinensis* (Turcz.) Baill (SC-extract) on the learning and memory impairment induced by Aβ_1–42_ in rats. Escape latencies were examined with the Morris water maze is hidden platform test (A~L). The escape latency (A~D), swimming distances (E~F), and average distance to the platform (I~L) during response acquisition and space exploration experiment session (phase 1) by the different groups (D). Representative swim traces of each group during the probe and extinction trials (M). *n* = 8–11 each. * *p* < 0.05; ** *p* < 0.01. Figure S2. Inter-correlation between short-chain fatty acids (SCFAs) concentrate and indexes. The size of the nodes in the dot plot shows the Spearman’s correlation coefficient, and the red and blue colors of the nodes represent positive and negative correlations, respectively. The marker of “×” means to be no significant for the correlation coefficient (False Discovery Rate adjusted *p*-value ≥ 0.05). Figure S3. The chromatographic charts of a representative sample, control solvent, and blank solvent for the colon content sample. Figure S4. The chromatographic charts of a representative sample, control solvent, and blank solvent for the serum sample. Figure S5. Calibration curves for each SCFAs in the colon content sample. Figure S6. Calibration curves for each SCFAs in the serum sample. Table S1. The comparison of protocols to measurement SCFA in the colon content and serum. Table S2. Methodological observation: Linearity (calibration curves). Table S3. Methodological observations: Repeatability, intra- and inter-day precision, and recovery (L, low-concentration quality control; M, medium-concentration quality control; H, high-concentration quality control; *n* = 3). Table S4. The comparison of SCFA concentration (μM) in the serum and colon content samples. Table S5. Selected ions, chromatographic, and mass spectrometric characteristics of the analytes for detection in MRM mode.
