# Supplementary material for: Determination and Comparison of Short-Chain Fatty Acids in Serum and Colon Content Samples: Alzheimer’s Disease Rat as a Case Study
Source: Molecules. 2020 Dec 4;25(23):5739. doi: 10.3390/molecules25235739 (PMC7729479; doi:10.3390/molecules25235739)
Supplement: Supplementary file 1 [file molecules-25-05739-s001.pdf]

Supplementary Materials

Figure S1. Effects of SC extract on the learning and memory impairment induced by Aβ1-42 in rats. Escape latencies were examined with the Morris water maze hidden platform test (A~L). The escape latency (A~D), swimming distances (E~F) and average distance to the platform (I~L) during response acquisition and space exploration experiment session (phase 1) by the different groups (D). Representative swim traces of each group during the probe and extinction trials (M). n = 8-11 each. * P < 0.05; ** P < 0.01.

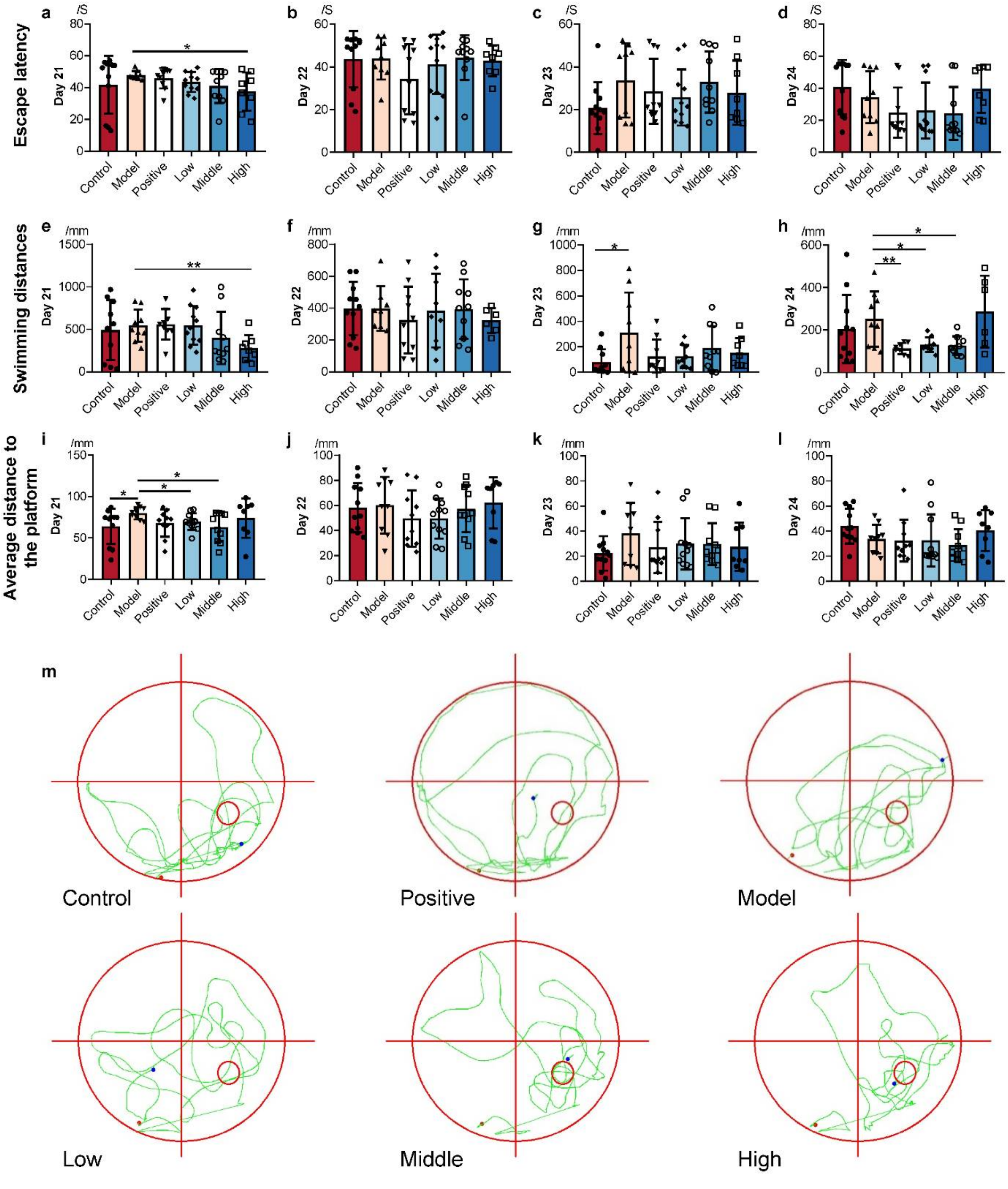

Figure S2. Inter-correlation between SCFAs concentrate and indexes. The size of the nodes in the dot plot shows the Spearman's correlation coefficient, and the red and blue colors of the nodes represent positive and negative correlations, respectively. The marker of "×" means to be no significant for the correlation coefficient (False Discovery Rate adjusted p-value ≥ 0.05).

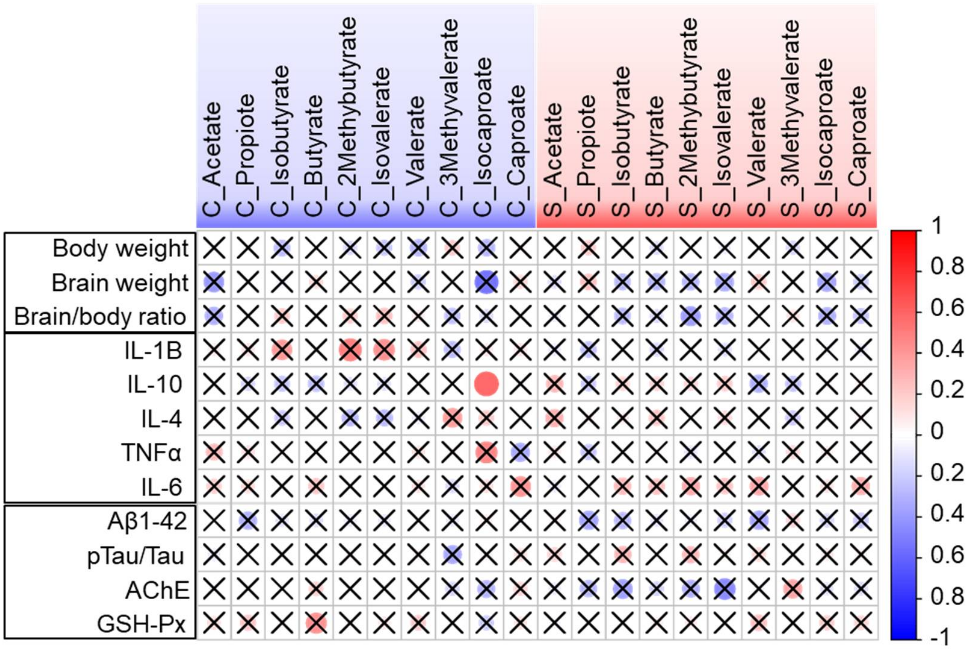

Figure S3. The chromatographic charts of representative sample, control solvent and blank solvent for colon content sample.

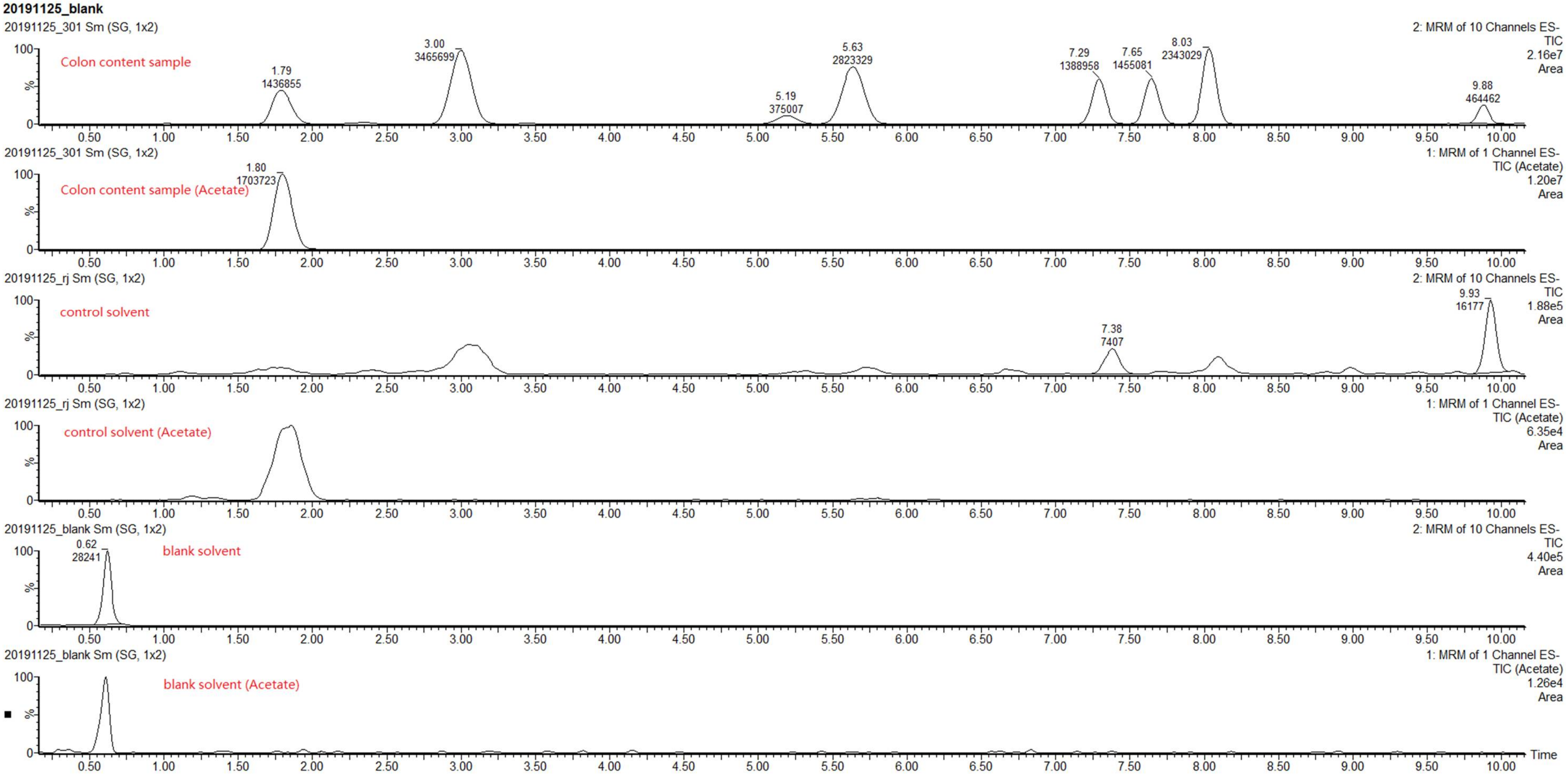

Figure S4. The chromatographic charts of representative sample, control solvent and blank solvent for serum sample.

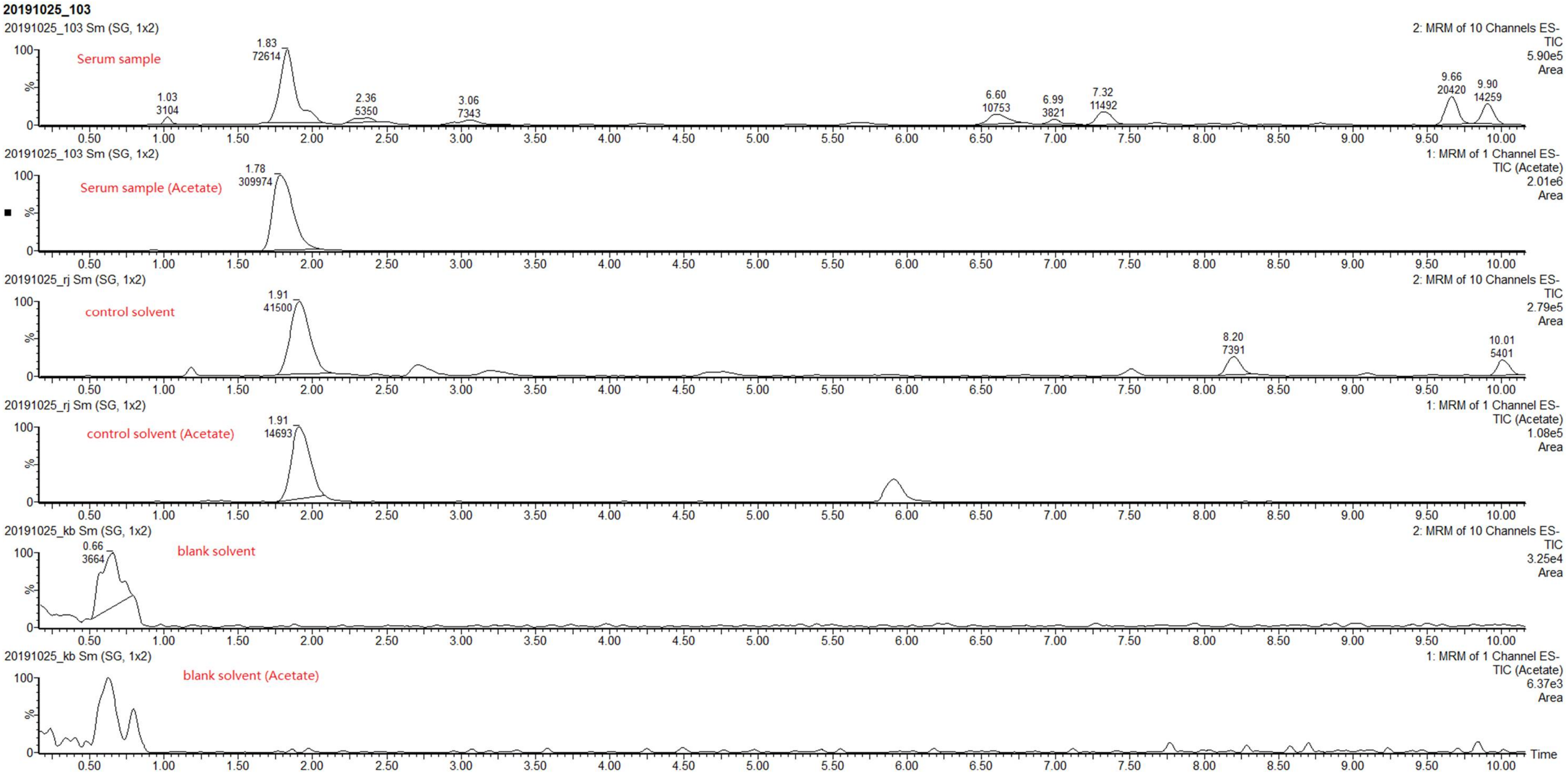

Figure S5. Calibration curves for each SCFA in colon content sample.

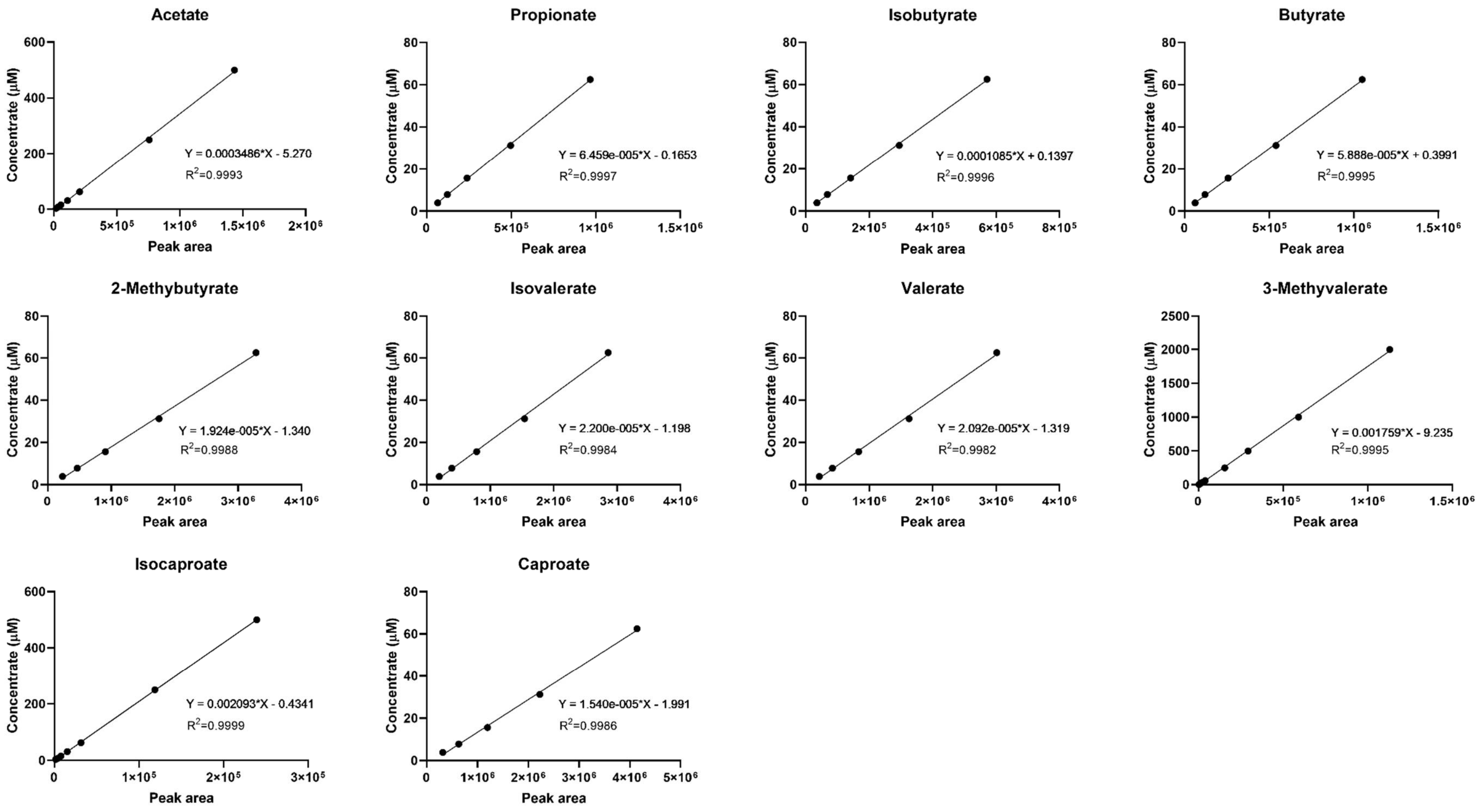

Figure S6. Calibration curves for each SCFA in serum sample.

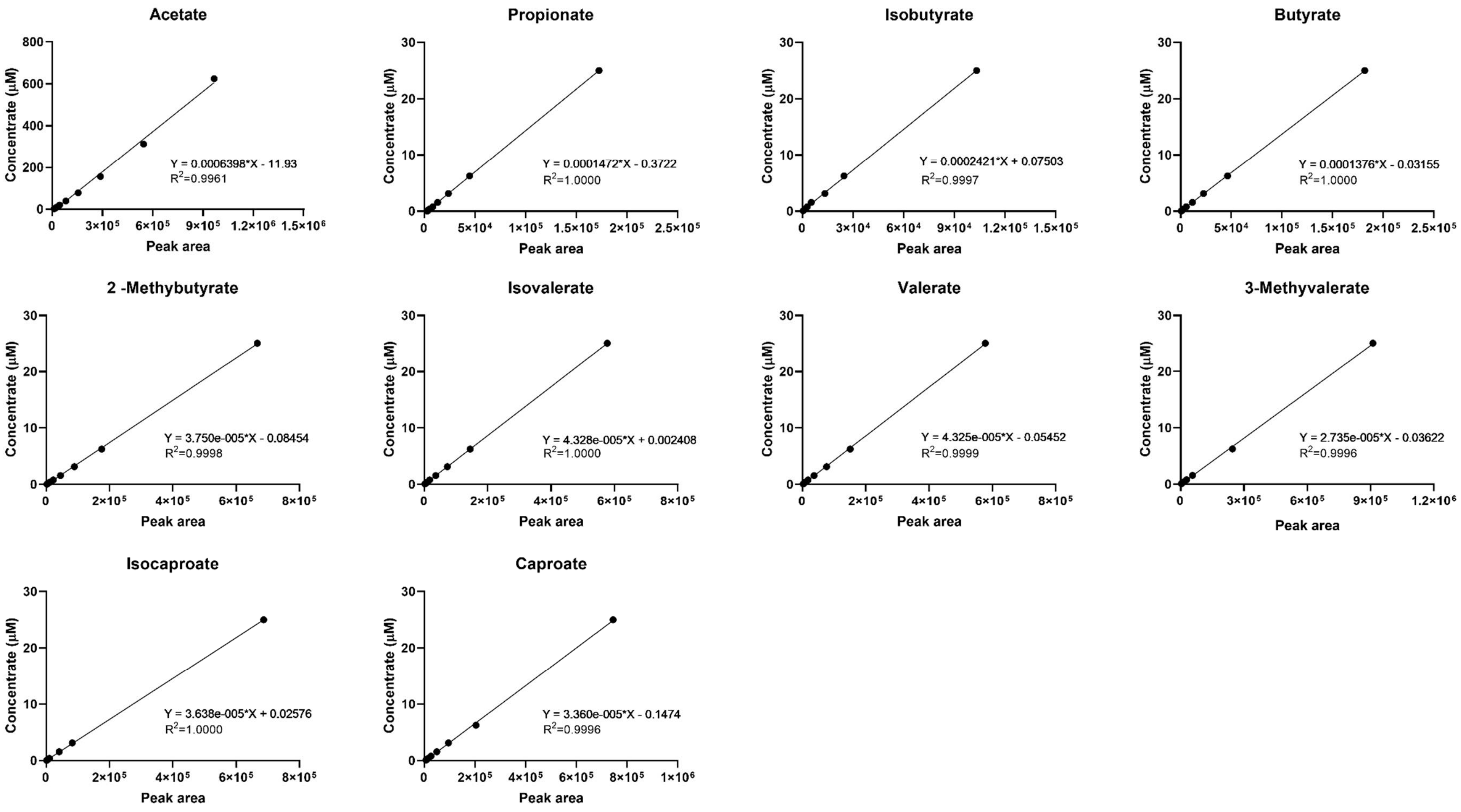

Table S1. The comparison of protocols to measurement SCFA in colon content and serum.

<table>
<tr><th colspan="2">Item</th><th>Colon content</th><th>Serum</th></tr>
<tr><td rowspan="4">Sample handling protocol</td><td>SCFAs extraction from samples</td><td>40 mg of freeze-drying of sample + 960 μL 30% acetonitrile → mixed by vortexing for 1 min → centrifuged at 13000 rpm for 10 min → collected 100 μL supernatant as SCFAs extract for derivatization</td><td>60 μL sample + 120 μL methanol → mixed by vortexing for 1 min → centrifuged at 5000 rpm for 10 min → collected 100 μL supernatant as SCFAs extract for derivatization</td></tr>
<tr><td>Derivative reagent (2 mL)</td><td>1 mL of 200 mmol/L 3-NPH + 200 μL of 600 mmol/L EDC + 800 μL 7.5% pyridine</td><td>1 mL of 100 mmol/L 3-NPH + 200 μL of 300 mmol/L EDC + 800 μL 3.75% pyridine</td></tr>
<tr><td>Derivatization</td><td colspan="2">100 μL SCFAs extract + 100μL derivative reagent → 40 ℃ for 30min</td></tr>
<tr><td>After chemical derivatization</td><td colspan="2">Cooled down at 4 ℃ → diluted to 1 mL with cold 10% acetonitrile → centrifuged at 13000 rpm for 10 min → 800 μL of the supernatant were collected as the test solution</td></tr>
<tr><td rowspan="2">LC/MS/MS conditions</td><td>UPLC conditions</td><td colspan="2">Instrumentation: ACQUITY UPLC H-Class system (Waters, USA)
Chromatographic column: a Waters Acquity BEH C18 column (2.1 × 50 mm, 1.7 m)
Mobile phases: 0.01 % formic acid in water (v/v) (A) and 0.01% formic acid in acetonitrile (v/v) (B)
Gradient: 0–3 min 15% B; 3–11 min 15-45% B; 11–12 min 45-99% B; 12–12.1 min 99-15% B; 12.1–14 min 15%
Column temperature: 40 ℃
Injection volume: 5 μL</td></tr>
<tr><td>MS conditions</td><td colspan="2">Instrumentation: a XEVO TQ-S mass spectrometer
Ionization source: the negative mode of Electrospray (ESI$^-$)
Capillary(kV): 2.7, Cone:27 V
Desolvation Temperatures: 250 ℃,
Desolvation gas flow: 600 L/Hr, Cone gas flow: 150 L/Hr</td></tr>
</table>

Table S2. Methodological observation: linearity.

Compound	Colon content				Serum			
	Calibration curves	R^2	Min	Max	Calibration curves	R^2	Min	Max
Acetate	Y = 0.0003486*X - 5.270	0.9993	3.9063	500	Y=6.398E-04*X-11.93	0.9961	1.2207	625
Propionate	Y = 6.459e-005*X - 0.1653	0.9997	3.9063	62.5	Y=1.472 E-04*X-0.3722	1.0000	0.0122	25
Isobutyrate	Y = 0.0001085*X + 0.1397	0.9996	3.9063	62.5	Y=2.421 E-04*X+0.075	0.9997	0.0977	25
Butyrate	Y = 5.888e-005*X + 0.3991	0.9995	3.9063	62.5	Y=1.376E-04*X-0.03155	1.0000	0.0244	25
2-Methybutyrate	Y = 1.924e-005*X - 1.340	0.9988	3.9063	62.5	Y=3.750E-05*X-0.08454	0.9998	0.0122	25
Isovalerate	Y = 2.200e-005*X - 1.198	0.9984	3.9063	62.5	Y=4.328E-05*X+0.002408	1.0000	0.0122	25
Valerate	Y = 2.092e-005*X - 1.319	0.9982	3.9063	62.5	Y=4.325E-05*X-0.05452	0.9999	0.0122	25
3-Methyvalerate	Y = 0.001759*X - 9.235	0.9995	3.9063	2000	Y=2.735E-05*X-0.03622	0.9996	0.0122	25
Isocaproate	Y = 0.002093*X - 0.4341	0.9999	3.9063	500	Y=3.638E-05*X+0.02576	1.0000	0.0122	3.125
Caproate	Y = 1.540e-005*X - 1.991	0.9986	3.9063	62.5	Y=3.360E-05*X-0.1474	0.9996	0.0122	25

Table S3. Methodological observations: repeatability, intra- and inter-day precision and recovery.

Sample	Compound	Repeatability (CV, %)			Intra-day Precision (CV, %)			Inter-day Precision (CV, %)			Recovery (%)		
		L	M	H	L	M	H	L	M	H	L	M	H
Colon content	Acetate	1.56	1.47	3.10	3.94	5.75	6.41	12.10	3.32	13.11	94.04	98.02	87.61
	Propionate	1.81	1.99	3.53	3.91	6.00	7.62	15.47	2.99	17.82	87.57	103.56	90.55
	Isobutyrate	2.91	3.16	3.43	4.40	7.23	7.68	16.18	2.62	17.77	86.83	112.00	93.99
	Butyrate	1.72	2.01	3.73	3.98	6.34	7.24	16.01	2.57	17.75	108.51	110.52	92.70
	2-Methybutyrate	1.27	2.28	3.15	4.81	7.21	5.92	13.96	2.09	9.51	93.79	114.67	97.41
	Isovalerate	1.31	1.85	3.26	3.21	6.17	6.25	11.88	2.27	10.86	93.31	113.04	93.79
	Valerate	2.12	1.62	3.19	4.79	5.78	6.09	15.17	2.35	12.22	95.46	111.01	97.54
	3-Methyvalerate	3.02	2.23	3.51	3.85	6.75	6.61	17.60	2.18	10.93	107.46	115.57	93.60
	Isocaproate	1.59	2.33	3.51	4.82	8.40	6.21	15.28	2.52	11.94	104.02	117.59	93.20
	Caproate	1.43	1.79	3.20	4.00	6.40	6.54	11.99	2.84	12.27	116.48	113.05	96.47
Serum	Acetate	1.82	1.81	0.23	1.64	1.19	1.08	1.30	1.55	1.27	108.22	101.82	93.25
	Propionate	3.12	4.33	2.85	6.49	2.38	1.91	7.81	4.64	2.04	106.21	100.56	93.05
	Isobutyrate	5.34	8.25	13.64	14.07	14.10	2.04	13.16	13.14	2.72	114.90	96.16	92.78
	Butyrate	5.77	4.20	5.71	6.09	11.99	2.46	5.52	8.10	2.25	109.38	105.68	92.42
	2-Methybutyrate	7.40	11.32	2.12	3.75	1.55	1.99	2.52	2.86	1.45	111.02	102.13	92.61
	Isovalerate	7.75	9.00	4.60	5.69	2.25	1.42	6.33	4.69	2.71	106.51	97.78	92.50
	Valerate	8.11	1.60	3.24	14.36	11.84	1.77	13.17	10.99	1.81	114.61	97.63	93.02
	3-Methyvalerate	9.48	5.43	6.83	8.06	2.70	1.87	7.72	4.07	2.19	108.05	90.06	92.77
	Isocaproate	9.72	14.02	9.89	1.17	2.21	2.47	3.88	4.12	3.11	108.34	98.05	93.56
	Caproate	9.96	10.92	6.99	12.57	3.35	1.87	11.24	7.69	3.22	108.35	97.63	93.35

Table S4. The comparison of SCFA concentration (μM) in serum and colon content samples.

Sample	Statistics	Acetate	Propionate	Isobutyrate	Butyrate	2-Methybutyrate	Isovalerate	Valerate	3-Methyvalerate	Isocaproate	Caproate
Colon content	Average	1293.18	401.05	56.34	296.66	30.50	40.98	61.83	1.26	74.79	18.20
	Standard deviation	505.50	176.28	24.10	139.60	11.78	16.43	32.69	1.27	138.10	11.23
	Variance	255533.53	31076.12	580.86	19489.30	138.81	269.96	1068.68	1.62	19072.73	126.19
	Range	2689.66	1006.87	108.46	651.50	50.48	72.85	171.26	5.91	716.34	49.94
	Minimum value	368.72	0.06	14.24	46.10	8.44	9.08	0.01	0.01	7.20	3.35
	Maximum value	3058.37	1006.93	122.70	697.60	58.92	81.93	171.27	5.92	723.55	53.29
	CV%	39.09	43.96	42.78	47.06	38.63	40.10	52.87	101.09	184.64	61.74
Serum	Average	69.46	0.23	0.24	0.63	0.23	0.13	0.02	0.03	0.89	0.40
	Standard deviation	81.50	0.40	0.32	0.63	0.33	0.18	0.01	0.07	0.62	0.37
	Variance	6642.33	0.16	0.10	0.39	0.11	0.03	0.00	0.01	0.39	0.14
	Range	295.05	3.04	2.57	2.31	2.57	0.99	0.05	0.60	2.67	1.89
	Minimum value	0.00	0.00	0.01	0.00	0.00	0.00	0.00	0.01	0.00	0.00
	Maximum value	295.05	3.04	2.58	2.31	2.57	0.99	0.05	0.62	2.67	1.89
	CV%	117.34	171.30	134.66	99.77	144.50	133.41	66.25	278.90	69.88	94.20

CV%: Coefficient of variation.

Table S5. Selected ions, chromatographic and mass spectrometric characteristics of the analytes for detection in MRM mode.

Compound	RT (min)	Parent ion (m/z)	Product ion(m/z)	Collision energy (eV)
Acetate	1.768	194.00	137.00	27
Propionate	2.929	208.07	137.00	25
Isobutyrate	5.086	222.10	179.00	19
Butyrate	5.536	222.10	152.00	22
2-Methybutyrate	7.206	236.10	137.00	27
Isovalerate	7.565	236.10	152.00	22
Valerate	7.956	236.10	193.00	20
3-Methyvalerate	9.321	250.10	137.00	28
Isocaproate	9.573	250.10	152.00	25
Caproate	9.813	250.10	207.10	22
